# Using AI to Differentiate Mpox From Common Skin Lesions in a Sexual Health Clinic: Algorithm Development and Validation Study

**DOI:** 10.2196/52490

**Published:** 2024-09-13

**Authors:** Nyi Nyi Soe, Zhen Yu, Phyu Mon Latt, David Lee, Ranjit Singh Samra, Zongyuan Ge, Rashidur Rahman, Jiajun Sun, Jason J Ong, Christopher K Fairley, Lei Zhang

**Affiliations:** 1 Artificial Intelligence and Modelling in Epidemiology Program Melbourne Sexual Health Centre Alfred Health Melbourne Australia; 2 School of Translational Medicine Faculty of Medicine, Nursing and Health Sciences Monash University Melbourne Australia; 3 Augmented Intelligence and Multimodal analytics (AIM) for Health Lab Faculty of Information Technology Monash University Melbourne Australia; 4 Melbourne Sexual Health Centre Alfred Health Melbourne Australia; 5 Department of Infectious Diseases Alfred Hospital Alfred Health Melbourne Australia; 6 Clinical Medical Research Center Children’s Hospital of Nanjing Medical University Nanjing China

**Keywords:** mpox, sexually transmitted infections, artificial intelligence, deep learning, skin lesion

## Abstract

**Background:**

The 2022 global outbreak of mpox has significantly impacted health facilities, and necessitated additional infection prevention and control measures and alterations to clinic processes. Early identification of suspected mpox cases will assist in mitigating these impacts.

**Objective:**

We aimed to develop and evaluate an artificial intelligence (AI)–based tool to differentiate mpox lesion images from other skin lesions seen in a sexual health clinic.

**Methods:**

We used a data set with 2200 images, that included mpox and non-mpox lesions images, collected from Melbourne Sexual Health Centre and web resources. We adopted deep learning approaches which involved 6 different deep learning architectures to train our AI models. We subsequently evaluated the performance of each model using a hold-out data set and an external validation data set to determine the optimal model for differentiating between mpox and non-mpox lesions.

**Results:**

The DenseNet-121 model outperformed other models with an overall area under the receiver operating characteristic curve (AUC) of 0.928, an accuracy of 0.848, a precision of 0.942, a recall of 0.742, and an *F*_1_-score of 0.834. Implementation of a region of interest approach significantly improved the performance of all models, with the AUC for the DenseNet-121 model increasing to 0.982. This approach resulted in an increase in the correct classification of mpox images from 79% (55/70) to 94% (66/70). The effectiveness of this approach was further validated by a visual analysis with gradient-weighted class activation mapping, demonstrating a reduction in false detection within the background of lesion images. On the external validation data set, ResNet-18 and DenseNet-121 achieved the highest performance. ResNet-18 achieved an AUC of 0.990 and an accuracy of 0.947, and DenseNet-121 achieved an AUC of 0.982 and an accuracy of 0.926.

**Conclusions:**

Our study demonstrated it was possible to use an AI-based image recognition algorithm to accurately differentiate between mpox and common skin lesions. Our findings provide a foundation for future investigations aimed at refining the algorithm and establishing the place of such technology in a sexual health clinic.

## Introduction

Mpox is a zoonotic viral infection caused by the monkeypox virus [1]. The infection originated from remote areas of Central and West Africa and was first reported in 1970 in the Democratic Republic of Congo. The disease typically presents with initial symptoms including fever, headache, rash, and lymph node enlargement [1,2]. It can also present as lesions, sores, or ulcers in the face, mouth, extremities, and anogenital areas [3]. Mpox shares many similarities with other pox-like infections, such as chickenpox, smallpox, and other bacterial and viral infections. Mpox is transmitted through close contact with infected individuals and the most recent cases have occurred predominantly in men who have sex with men, especially those with multiple and anonymous sexual partners. Although mpox is generally self-limiting, delayed diagnosis could lead to transmission within communities and increase the risk of complications in immunocompromised individuals, children, and pregnant women [4].

The 2022 global mpox outbreak significantly impacted health facilities because it required additional infection prevention and control measures including personal protective equipment to prevent the spread of infection to staff [5,6]. Moreover, suspected mpox cases had to be separated from main clinic patients to mitigate the risk of infection to others, and so the processes in the clinic were modified and slowed [7]. If it were possible to identify suspected mpox cases before their visit, it would benefit the workflow of clinics.

Recently, artificial intelligence (AI)–assisted medical diagnosis has emerged as a promising research area in the health care sector [8]. AI has been used in medical imaging to assist radiologists in identifying COVID-19–related abnormalities in chest x-rays and computed tomographic scans [9]. AI algorithms can diagnose skin lesions such as melanoma with high accuracy [10-12] and have seen increasing use in risk prediction [13,14] and early diagnosis of sexually transmitted infections (STIs) [15]. To our knowledge, many studies [16-20] have been conducted to examine the performance of tools using AI to diagnose mpox. These studies were designed to differentiate mpox from chickenpox, smallpox, cowpox, and measles. While these studies provided valuable insights, their application may be limited in today’s clinical setting where cowpox is rare and smallpox has been eliminated. In the context of a sexual health clinic, a diverse range of skin lesions, including STIs such as genital herpes, genital warts, syphilis, and molluscum contagiosum. Nonsexually transmitted skin conditions such as dermatosis and other inflammatory skin diseases are also commonly seen.

We aimed to develop an AI-assisted diagnostic tool and evaluate its ability to distinguish mpox from other common skin lesions (STIs and non-STIs) in a sexual health clinic setting. If proven accurate, such a tool could offer a preliminary screening capability through a web-based platform, potentially aiding in the early detection of mpox cases and timely initiation of treatment. Furthermore, its integration into clinical workflows could streamline the triage process and enhance patient management in the clinic.

## Methods

### Data Collection

Melbourne Sexual Health Centre (MSHC) conducted this study using the checklist for Artificial Intelligence in Medical Imaging (CLAIM) [21]. Since 2010, we have collected clinical images of STIs and non-STI skin lesions. In 2022 we added mpox lesion images to our database. The MSHC data set included 86 mpox and 1565 non-mpox images. We also implemented web scraping techniques on Google and Bing (Microsoft) search engines with different terms (“monkeypox,” “Monkey Pox,” “MPX,” and “Mpox”) to collect public domain or Creative Commons mpox images. This enabled us to collect 271 mpox images which required clinicians’ validation. Both MSHC and internet-sourced data sets contained uncropped original images. Moreover, we included 278 mpox images from the Kaggle data set [22] used in previous studies [16-18] for model evaluation. This data set consists of anonymized skin lesion images without any associated patient information, precluding the comparison of patient characteristics across different data sets. The images from the Kaggle data set had already undergone preprocessing and their lesion areas were cropped for evaluation.

### Inclusion and Exclusion Criteria

The study included images collected from the MSHC attendees who provided written informed consent for the use of their image for research purposes and who were diagnosed with mpox, genital herpes, genital warts, primary or secondary syphilis, molluscum contagiosum, dermatosis, or healthy skin. The web-scraped mpox images were included if they were open source and could be verified by MSHC clinicians. Images not meeting these criteria were excluded.

### Data Labeling

Two experienced sexual health physicians independently labeled images that did not have diagnoses and ensured they were correct by reviewing the clinical notes, and laboratory results from the Clinical Patient Management System. For the images from the Kaggle data sets, the diagnoses were also verified visually by the experts. Any discrepancies in image diagnosis between the 2 clinicians were reviewed by a third clinician. If consensus on the diagnosis could not be reached between the 3 clinicians, the image was discarded. Our study focused on distinguishing mpox from other common skin lesions, thus, labeled images were organized into mpox and non-mpox folders. Table 1 shows the number of images for each disease class.

**Table 1:** Distribution of images across diagnostic classes.

Class and diseases		# Original images, n (%)	# Augmented images, n	# Total images, n
**Mpox**				1537
	Mpox	635 (28.86)	902	
**Non-mpox**				1565
	Dermatosis	215 (9.77)	0	
	Genital herpes	250 (11.36)	0	
	Molluscum contagiosum	200 (9.09)	0	
	Healthy skin	250 (11.36)	0	
	Primary syphilis	200 (9.09)	0	
	Secondary syphilis	250 (11.36)	0	
	Genital warts	200 (9.09)	0	
Total		2200 (100.00)	902	3102

### Data Processing

We manually checked all images to ensure they had no identifiable features (eg, face, tattoos, and birthmarks). We then removed 14 duplicated and low-resolution images from the whole data set by using the difPy Python package (Python Software Foundation) [23]. As part of the image preprocessing pipeline, all original images were resized to a fixed resolution of 320×320 pixels and converted to JPEG format to ensure uniformity and facilitate subsequent processing.

### Data Partition

**Figure 1:**
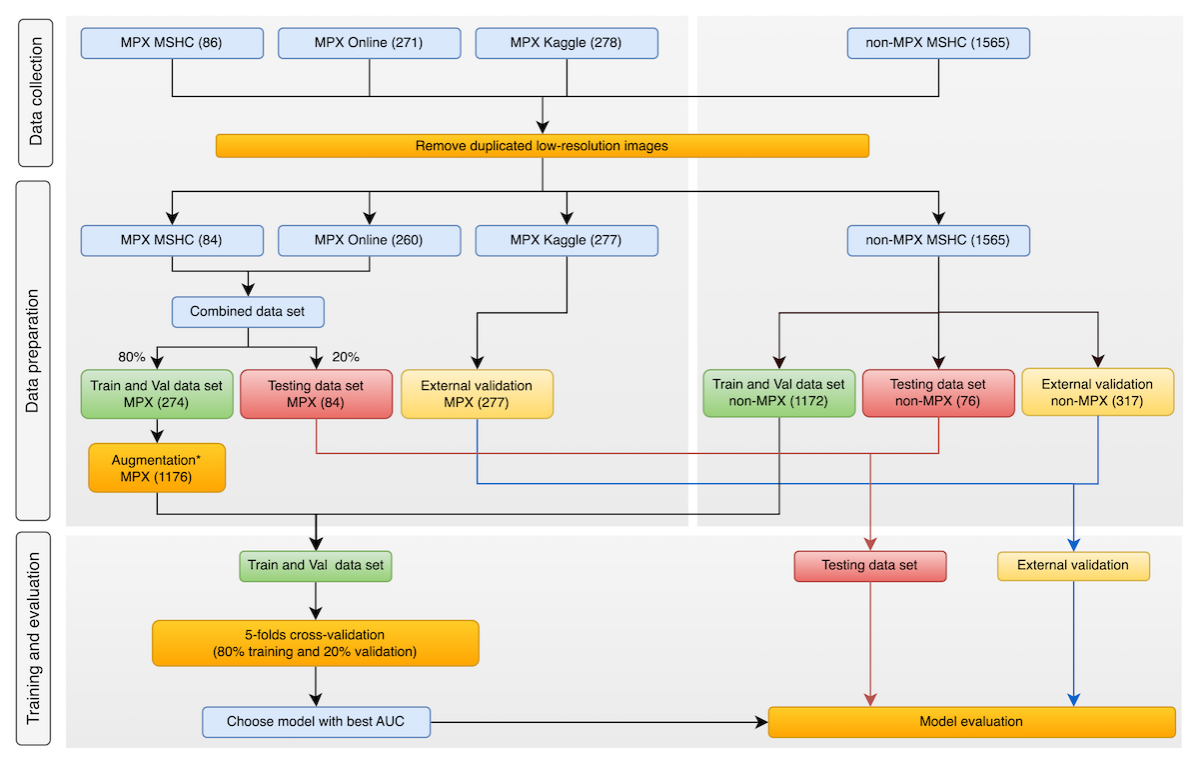
Workflow of the AI-based mpox classification model. AI: artificial intelligence; AUC: area under the receiver operating characteristic curve; MPX: mpox; MSHC: Melbourne Sexual Health Centre.

We divided the main data set into 3 subsets for training, testing, and external validation (see Figure 1). Except for those from the Kaggle data set, all mpox images were randomly divided into the “training and validation” data set (n=274, 80% of images) and the “testing” data set (n=70, 20% of images). The 277 mpox images from the Kaggle data set were reserved for “external validation.” Similarly, non-mpox images were also randomly divided into “training and validation,” “testing,” and “external validation,” with 1172, 76, and 317 images, respectively.

### Image Augmentation

The training and validation data set was imbalanced, with 274 mpox images but 1172 non-mpox images. To address this imbalanced data set [24], we applied data augmentation to increase the number of images in the mpox data set [25]. This included rotating images, cropping areas, zooming in or out, flipping horizontally or vertically, and adjusting brightness and contrast (see Table S1 in Multimedia Appendix 1 for data augmentations). We generated 902 new images for mpox with augmentation, resulting in a final training and validation data set of 1176 mpox and 1172 non-mpox images. Testing and external validation data sets were not augmented and the final data sets contained 146 and 594 images, respectively (see Table 1 and Figure 1).

### Model Training and Validation

**Figure 2:**
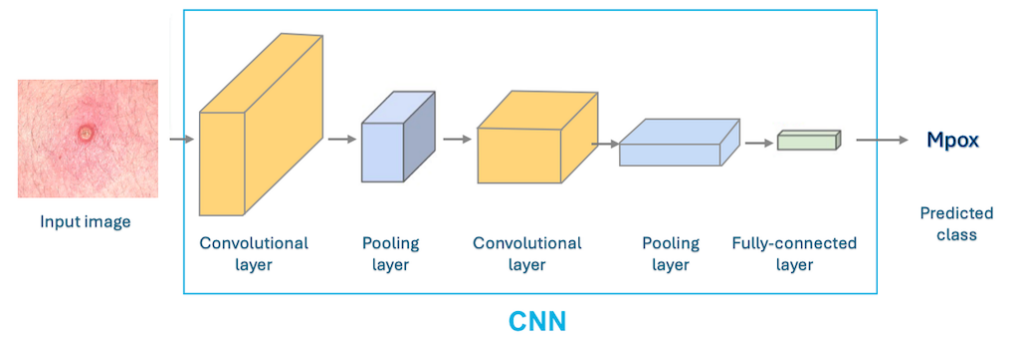
Overview of convolutional neural network (CNN) architecture.

We adopted a transfer learning approach to develop the binary image classification model. This approach involved using the pretrained model on a large data set and fine-tuning it on a smaller target data set, making it effective in training a deep model with fewer images [26,27]. During transfer learning, we froze the weights of all backbone layers and replaced the last fully connected layer with a new classification layer. We only trained it with random weights thereafter. We experimented with 6 pretrained deep neural network architectures with different-sized parameters—MobileNet-V2 [28], ShuffleNet-V2 [29], DenseNet-121 [30], ResNet-18 [31], ResNet-34 [31], and Swin-Transformer [32] (see Figure 2). The size of the parameters can be seen in Table S2 in Multimedia Appendix 1.

A 5-fold cross-validation was performed on the training and validation data sets, to enhance the model’s robustness and generalizability. We trained and validated each pretrained model 5 times with different subsets, and the results were averaged for better performance evaluation. We trained an AI image classification model using a pretrained model backbone, Adam optimizer, cross-entropy loss function, a batch size of 72, a dropout rate of 0.2, an image size of 320×320 pixels, 150 epochs, and a learning rate of 3e^-4^. We implemented model training with PyTorch (Meta AI) on a Tesla T4 GPU machine. The model training time ranged from 3 to 4 hours for smaller models like MobileNet-V2 and ShuffleNet-V2, while larger models like Swin-Transformer took up to 5 to 6 hours.

### Performance Evaluation Metrics

The model performance for distinguishing between mpox and non-mpox images was evaluated with various metrics including area under the receiver operating characteristic curve (AUC), accuracy, precision, recall, and *F*_1_-score. AUC measures the model’s ability for binary classification, ranging from 0 to 1, where 1 indicates perfect classification [33-35]. The equation and definition of the performance metrics are shown in Table S3 in Multimedia Appendix 1. The scikit-learn Python package (version 1.2.0) was used to calculate all metrics [36].

### Region of Interest Approach to Improve Model Performance

In this study, the original images from MSHC were used for the testing data set without any cropping of the lesion areas, whereas the mpox images from the Kaggle data set were precropped for the area of interest in the external validation data set. The performance of the model was compared between the testing and external validation data sets. Following this, a region of interest (ROI) approach was applied to the testing data set, whereby the lesion areas were cropped from the original images, and the model’s performance was reevaluated. The efficacy of this approach was visualized using gradient-weighted class activation mapping (Grad-CAM), which enabled the assessment of the model’s performance before and after the ROI approach.

### Model External Validation

We first evaluated all 6 models with a testing data set that had never been used for model training and calculated the performance metrics. To further test the generalizability and robustness of those models, we evaluated their performance with an external validation data set containing unseen images from the Kaggle data set, which was commonly used in previous studies (Figure 1). Then, we computed the confusion matrices for the best models on testing and external validation data sets to analyze the model’s correct and incorrect predictions. Finally, we conducted the visual analysis on a set of sample prediction outputs from the best model using Grad-CAM [33]. We generated the image with a heatmap where the most important region for the predicted class was highlighted. In other words, the features of the images used for prediction were highlighted with color gradients from the most important to the least important areas, helping to understand the model’s behavior and to identify the error for wrong predictions.

### Ethical Considerations

The study protocol received ethics approval from the Alfred Hospital Ethics Committee (683/22). All research activities were carried out in compliance with relevant ethical principles and guidelines. To protect participant privacy, all data underwent a deidentification process before being used for model development and analysis.

## Results

### Overview

Table 1 shows the number of images with mpox and non-mpox conditions in the data set. The mpox images accounted for 29% (635/2200) of the original images while other non-mpox images included conditions that individually accounted for between 9% (200/2200) and 11% (250/2200) of the data set. Figure 1 shows the processes for how the final data sets were created and the source of images for the final data sets.

**Figure 3:**
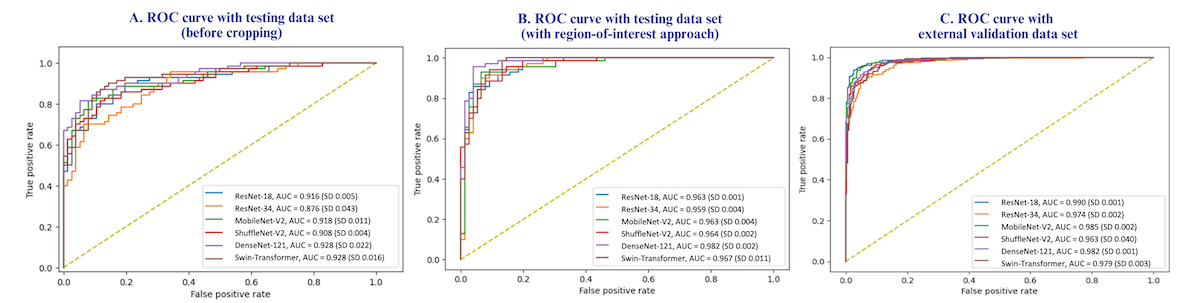
AUC comparison of 6 different models on testing and external validation data set. AUC: Area Under the Receiver Operating Characteristic Curve; ROC: Receiver Operating Characteristics Curve.

Table 2 shows key performance metrics for the 6 different models, including AUC, accuracy, precision, recall, and *F*_1_-score. These metrics are presented for the testing data set both before cropping and after applying the ROI approach, as well as for the external validation data set. Additionally, Figure 3 shows the visual comparison of the AUC for 6 different models across different data sets.

**Table 2:** Model evaluation: performance metrics across 5 folds.

Model		AUC^a^, mean (SD)	Accuracy, mean (SD)	Precision, mean (SD)	Recall, mean (SD)	*F*_1_-score, mean (SD)
**Model evaluation on testing data set (before cropping)**						
	MobileNet-V2	0.918 (0.011)	0.832 (0.022)	0.942 (0.025)	0.686 (0.054)	0.792 (0.033)
	ShuffleNet-V2	0.908 (0.004)	0.820 (0.014)	0.964 (0.022)	0.618 (0.049)	0.752 (0.035)
	DenseNet-121	0.928 (0.022)	0.848 (0.041)	0.942 (0.013)	0.742 (0.024)	0.834 (0.018)
	ResNet-18	0.916 (0.005)	0.828 (0.008)	0.934 (0.026)	0.688 (0.016)	0.79 (0.010)
	ResNet-34	0.876 (0.043)	0.798 (0.015)	0.900 (0.000)	0.618 (0.011)	0.734 (0.005)
	Swin-Transformer	0.928 (0.016)	0.798 (0.008)	0.926 (0.019)	0.622 (0.022)	0.746 (0.011)
**Model evaluation on testing data set (with region of interest approach)**						
	MobileNet-V2	0.963 (0.004)	0.907 (0.006)	0.949 (0.006)	0.851 (0.016)	0.898 (0.008)
	ShuffleNet-V2	0.964 (0.002)	0.871 (0.003)	0.924 (0.008)	0.797 (0.012)	0.856 (0.005)
	DenseNet-121	0.982 (0.002)	0.951 (0.003)	0.956 (0.000)	0.940 (0.006)	0.948 (0.003)
	ResNet-18	0.963 (0.001)	0.901 (0.008)	0.922 (0.018)	0.869 (0.012)	0.894 (0.008)
	ResNet-34	0.959 (0.004)	0.882 (0.009)	0.918 (0.007)	0.829 (0.017)	0.871 (0.011)
	Swin-Transformer	0.967 (0.011)	0.862 (0.013)	0.931 (0.002)	0.769 (0.027)	0.842 (0.018)
**Model evaluation on external validation data set**						
	MobileNet-V2	0.985 (0.002)	0.937 (0.007)	0.911 (0.019)	0.959 (0.017)	0.934 (0.007)
	ShuffleNet-V2	0.963 (0.040)	0.923 (0.005)	0.925 (0.009)	0.910 (0.003)	0.917 (0.005)
	DenseNet-121	0.982 (0.001)	0.926 (0.003)	0.906 (0.006)	0.939 (0.006)	0.922 (0.003)
	ResNet-18	0.990 (0.001)	0.947 (0.006)	0.934 (0.009)	0.953 (0.009)	0.943 (0.006)
	ResNet-34	0.974 (0.002)	0.908 (0.008)	0.887 (0.013)	0.920 (0.006)	0.903 (0.008)
	Swin-Transformer	0.979 (0.003)	0.912 (0.007)	0.944 (0.005)	0.862 (0.012)	0.901 (0.009)

^a^AUC: area under the receiver operating characteristic curve.

### Evaluation of Model Performance on the Testing Data Set (Before Cropping)

Evaluation of the performance of 6 different models with the testing data set (before cropping) showed that the DenseNet-121 outperformed the other models in terms of overall AUC (0.928, SD 0.022), accuracy (0.848, SD 0.041), precision (0.942, SD 0.013), recall (0.742, SD 0.024), and *F*_1_-score (0.834, SD 0.018; Table 2).

### Evaluation of the Model With the ROI Approach on the Testing Data Set

We applied the ROI approach to the testing data set and conducted a reevaluation of the models (Table 2). This approach significantly improved the performance of all 6 models for image classification. The AUC score of MobileNet-V2 increased from a mean of 0.918 (SD 0.011) to a mean of 0.963 (SD 0.004), ShuffleNet-V2 from a mean of 0.908 (SD 0.004) to a mean of 0.964 (SD 0.002), ResNet-18 from a mean of 0.916 (SD 0.005) to a mean of 0.963 (SD 0.001), ResNet-34 from a mean of 0.876 (SD 0.043) to a mean of 0.959 (SD 0.004), and Swin-Transformer from a mean of 0.928 (SD 0.016) to a mean of 0.967 (SD 0.011). The AUC score of the best-performing DenseNet-121 model increased from a mean of 0.928 (SD 0.022) to mean of 0.982 (SD 0.002) on the testing data set following the adoption of the ROI approach.

### Evaluation of Model Performance on the External Validation Data Set

We evaluated the performance of these models with the external validation data set. The findings in Table 2 showed that the ResNet-18 performed best on this data set, achieving an AUC of mean 0.990 (SD 0.001), an accuracy of 0.947 (SD 0.006), a precision of 0.934 (SD 0.009), a recall of 0.953 (SD 0.009), and an F-score of 0.943 (SD 0.006). DenseNet-121 also achieved high performance with an AUC of 0.982 (SD 0.001) on the external validation data set.

### Confusion Matrix Results for DenseNet-121 and ResNet-18 Models

**Figure 4:**
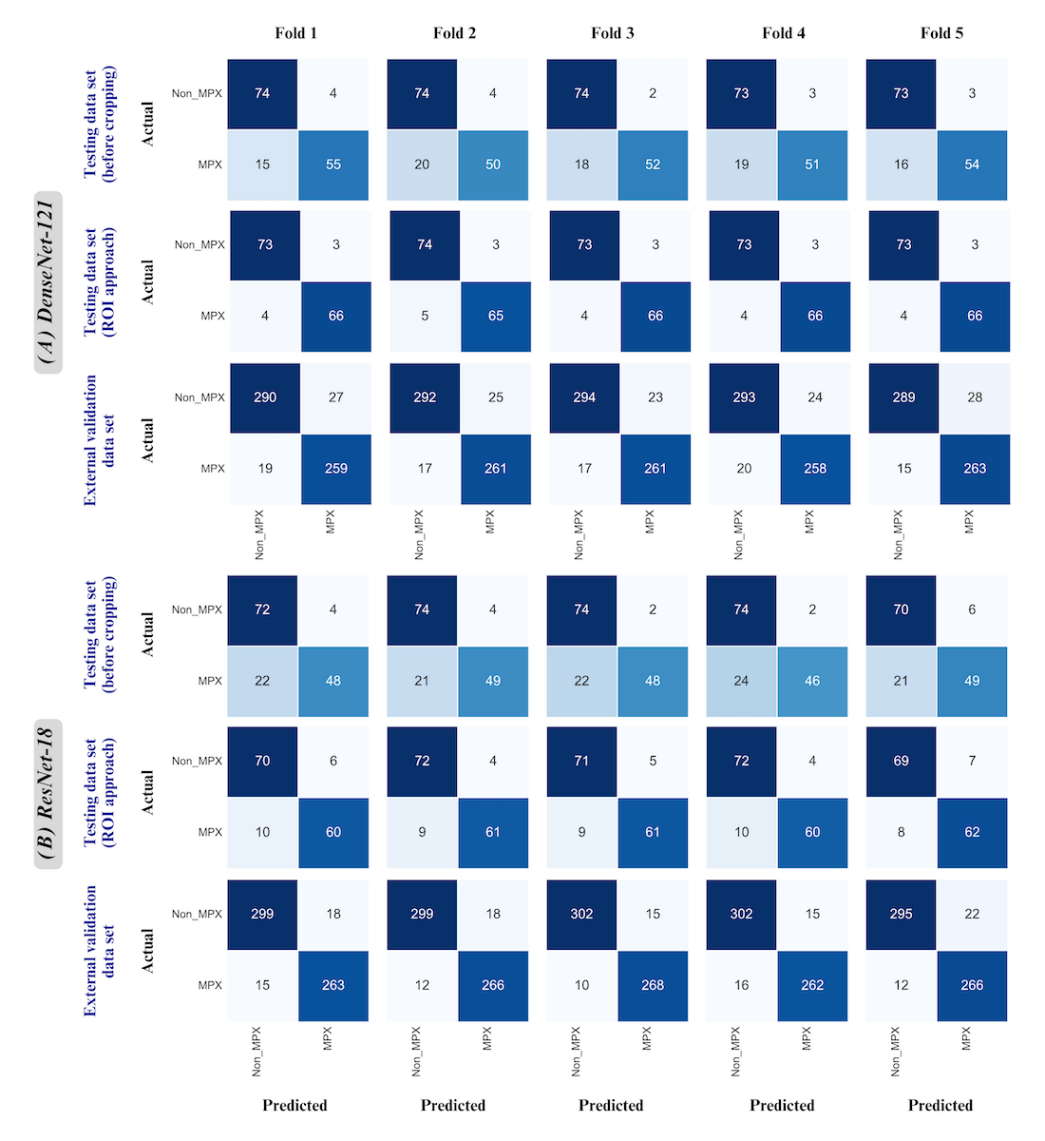
Confusion matrix for best models (5-fold cross-validations). ROI: region of interest.

Figure 4 shows the confusion matrixes for 5 folds for the DenseNet-121 and ResNet-18 models. In the testing data set (before cropping), the confusion matrix of the best fold in DenseNet-121 showed that 55 out of 70 (79%) mpox and 74 out of 77 (96%) non-mpox images were correctly classified (Figure 4A). After applying the ROI approach, the confusion matrix of the best fold in DenseNet-121 showed that 66 out of 70 (94%) mpox images were correctly classified which represents a notable increase from 79% (55/70) before applying this approach (Figure 4A). In the external validation data set, the confusion matrix of the best fold in ResNet-18 showed that 283 out of 268 (95%) mpox and 302 out of 312 (97%) non-mpox images were correctly identified by the model (Figure 4B).

### Visual Analysis With Grad-CAM

**Figure 5:**
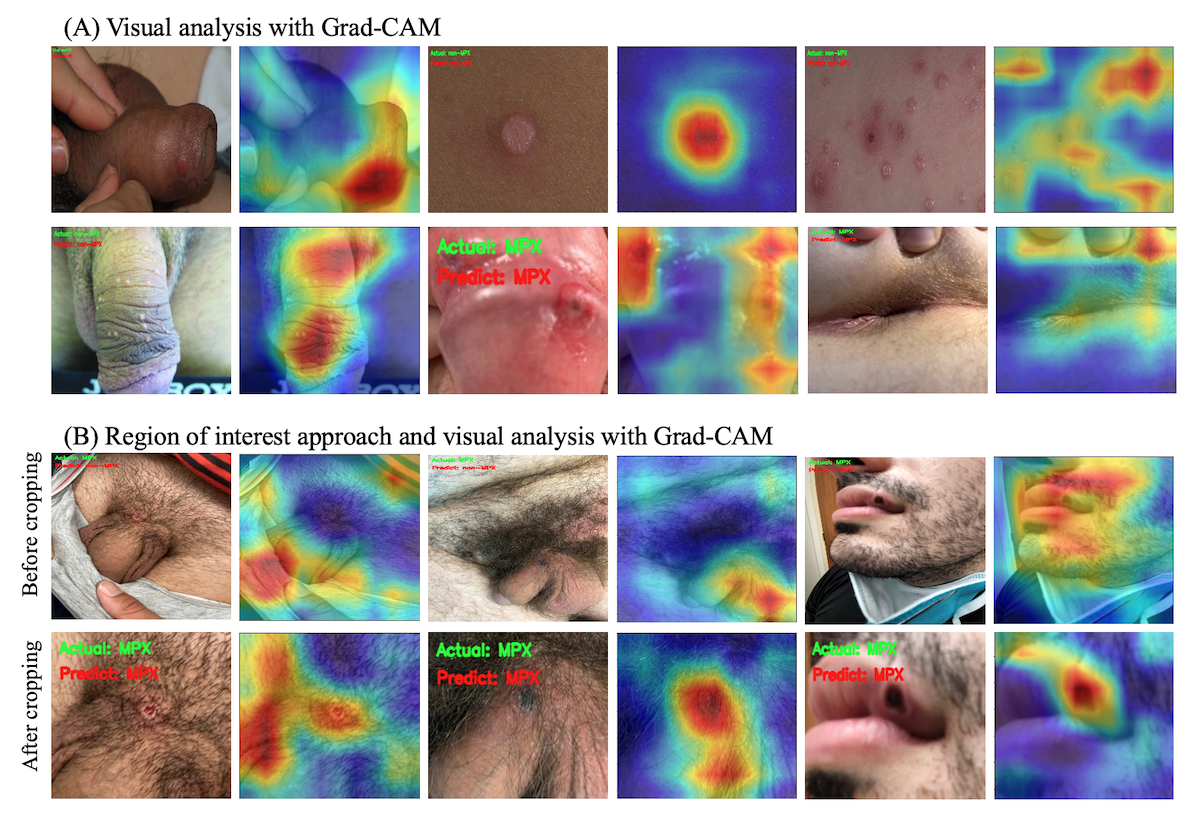
Visual analysis on predicted image class from DenseNet-121. (A) The original image (left) and the corresponding heatmap image (right) generated by Grad-CAM were shown and the heatmap highlighted regions of the image that the model used to make its prediction. (B)The first row showed the original image, and the second row showed the same image after cropping ROI, showing the accuracy of the model improved after the ROI approach. Grad-CAM: gradient-weighted class activation mapping; ROI: region of interest.

Figure 5 shows the visual analysis of the lesion images with Grad-CAM. The visual analysis using Grad-CAM on random images from the testing data set (before cropping) showed inaccuracies in the areas of importance for predicted diagnosis (see Figure 5B, first row). We observed false detections occurring in the background rather than the lesion areas of the images from the testing data set. After applying the ROI approach to the testing data set, the model’s accuracy in detecting lesion areas showed a significant improvement (Figure 5B, second row).

## Discussion

### Principal Findings

Previous studies [16-18] have shown AI tools can differentiate mpox from non-STI lesions such as measles, smallpox, and chickenpox. Our study added to this by demonstrating that AI can differentiate mpox from images of common skin lesions including STIs and non-STIs collected from a sexual health clinic. We showed that the DenseNet-121 model performed most accurately compared to other models although the differences were small. We also demonstrated that the accuracy of the image classification models could be significantly improved by cropping the main ROI lesion areas from the original image prior to feeding it to the model.

Our study found similar results to other published studies that used AI-based image recognition algorithms to detect mpox cases among symptomatic clinic attendees. The DenseNet-121 model outperformed the other models, achieving a 0.951 accuracy on the testing data set with 147 MSHC mpox images confirmed by laboratory diagnosis. In addition, our DenseNet-121 model also demonstrated a 0.926 accuracy on the external validation data set consisting of 544 mpox images from the Kaggle data set. This external validation data set was previously used in other studies [16-18]. Our model’s performance was similar to those used in previous studies. The study by Akin et al [18] achieved a 0.980 accuracy with ResNet-18 for binary classification between mpox and other lesions. Similarly, Shams et al [16] achieved an 0.830 accuracy with ResNet-50 for the same classification task. This higher accuracy was possibly due to the limited variety of lesion images in the testing data sets, consisting of 45 and 56 images, respectively. Another study by Islam et al [17] achieved 0.790 accuracy with ShuffleNet-V2 for multiple classifications among 6 different lesions, with a testing data set of 158 images. However, it is important to note that our findings could not be directly compared with these studies because they used smaller testing data sets, different lesion types, and different model architectures.

There are a number of advantages to our study over other published studies. First, our study was the first one to demonstrate the potential for developing an AI tool to differentiate mpox from common skin lesions from images collected from a sexual health clinic. Second, we used a significantly larger data set compared to previous AI-based mpox classification studies [16-18]. Our data set included 2200 images, in contrast to previous studies’ data sets, which included 804 and 228 images. Third, our study included the clinic images in addition to the open-source images from the web-scraping method. All diagnoses of the images from MSHC were confirmed with laboratory tests and clinically by well-experienced sexual health physicians. Finally, we used a 5-fold cross-validation process to improve the robustness and generalizability of our models.

Our study demonstrated the crucial role of preprocessing techniques, specifically focused on cropping the ROI lesion areas, within the context of differentiating mpox from non-mpox lesions. As exemplified by the incorrectly classified cases in Figure 5B, in the first row, confusing backgrounds such as fabrics or hairy skin patterns appeared to contribute to inaccurate model predictions prior to the ROI approach. Additionally, when the main lesion areas comprised very small regions relative to the entire image area, the model struggled to focus on the relevant features. By cropping the images to solely focus on the lesion areas, the model was able to better recognize the lesion features, resulting in improved accuracy. Our study highlights the importance of focusing on the lesion area with minimum background in achieving optimal performance of the AI model. This also underscores the importance of user education and training to ensure that users are able to apply them effectively when using the model in a clinical setting. Nonetheless, it could be a challenge when end users submit images without preprocessing in a real-world setting. It emphasizes the need to integrate image preprocessing functions into the user interface of the tool during deployment; however, it was out of the scope of our study. Overall, our findings provide important insights into the development and optimization of image classification models in medical applications.

There are substantial potential applications of this AI-assisted diagnosis tool. First, this AI-assisted diagnosis tool could be integrated with existing health care platforms, such as patient booking systems, to identify suspected mpox cases among clinic attendees even before they attend the service. Early identification of infectious cases could help manage clinic workflows through appropriate infection control processes. Moreover, the tool could be integrated into the clinic’s web-based services, allowing patients to check their symptoms and we could prioritize health services for those identified as mpox and also warn individuals of their risk of transmission. Furthermore, this tool could be integrated into a mobile app for point-of-care services, especially in low-resource health care settings where access to medical professionals may be limited. The mobile app could potentially provide real-time assistance to rural health care providers, enabling more efficient and cost-effective diagnosis. However, it is important to consider the ethical implications associated with the use of AI-assisted diagnostic tools, including concerns about data privacy and unintended consequences. It is necessary to develop appropriate guidelines and frameworks to mitigate these concerns.

Our study had several limitations. First, we used augmentation methods to multiply mpox classes to solve the imbalanced data which might affect the model performance during the training and validation process. Second, we included web-scraped mpox images and this limitation of not being able to confirm their diagnosis with laboratory tests was addressed by having them reviewed and visually diagnosed by sexual health physicians. Third, although our models yielded promising results, they also highlighted opportunities for improvement. The evaluation of model performance showed that there were some misclassifications between classes and false detection areas during visual analysis with Grad-CAM. Given these findings, further research could explore whether increasing the data set size and developing more customized deep learning approaches rather than relying on fine-tuning and ROI cropping methods might improve the model performance. Furthermore, while our models demonstrated differentiation between mpox and non-mpox, it has not been validated in a real-world clinical setting due to the declining number of mpox cases. Our study’s aim was primarily to demonstrate the potential use of AI tools to differentiate skin lesions and provide a foundation for future research to test the AI model in real-world scenarios. Finally, we did not use any epidemiological or clinical history data that may also improve the model. For example, currently, there are no new cases of mpox in Australia and so the low underlying incidence of mpox would be likely to influence the accuracy of the models. Moreover, the integration of demographic and sexual behavioral data, such as whether an individual identifies as men who have sex with men and number of sexual partners, could potentially improve the model’s performance.

### Conclusions

In this study, our study demonstrated the potential use of an AI-based image recognition algorithm to differentiate between mpox and common skin lesions. The model with a high accuracy of 95% indicates its potential benefit from clinic workflow management and prevention of the transmission of mpox infection. However, further research is needed to improve the performance of the model and validate its effectiveness in real-world clinical settings. Overall, the successful development and evaluation of this AI-based image recognition algorithm offer a promising approach to improving diagnostic accuracy and efficiency in sexual health clinics.

## Appendix

Multimedia Appendix 1 Data augmentation to address the imbalanced dataset, comparison of pretrained models and their approximate trainable parameters and model size, and performance metrics for classification model: equation and definition.

## Abbreviations

AI: artificial intelligence
AUC: area under the receiver operating characteristic curve
CLAIM: checklist for Artificial Intelligence in Medical Imaging
MSHC: Melbourne Sexual Health Centre
ROI: region of interest
STI: sexually transmitted infection

## Data Availability

The data sets generated and analyzed during this study are not publicly available due to privacy and consent limitations for images collected from the Melbourne Sexual Health Centre (MSHC), but are available from the corresponding author on reasonable request. The external validation data set used in this study is publicly available on Kaggle, titled “Monkeypox Skin Image Dataset.”
