# Supplementary material for: Using AI to Differentiate Mpox From Common Skin Lesions in a Sexual Health Clinic: Algorithm Development and Validation Study
Source: J Med Internet Res. 2024 Sep 13;26:e52490. doi: 10.2196/52490 (PMC11437223; doi:10.2196/52490)
Supplement: Multimedia Appendix 1 [file jmir_v26i1e52490_app1.docx]

**Table S1.** Data augmentation to address the imbalanced dataset.

| **Augmentation methods** | **Settings** |
| --- | --- |
| Rotating image with random degree | ranged [-20, 20], probability = 1.0 |
| Cropping random 80% areas of image | probability = 0.5 |
| Zooming in and out | ranged [0.8, 1.4], probability = 0.5 |
| Flipping horizontally or vertically | probability = 0.5 |
| Adjusting brightness and contrast with random factor | ranged [-0.5, 1.5]). |

**Table S2.** Comparison of pretrained models and their approximate trainable parameters and model size.

| **Model Size** | **Pretrained Model** | **Parameters (in million)** |
| --- | --- | --- |
| Small | *MobileNet-V2* | 3.5 |
|  | *ShuffleNet-V2* | 2.2 |
|  | *DesnseNet-121* | 8.0 |
| Medium | *ResNet-18* | 11.7 |
|  | *ResNet-34* | 21.8 |
| Large | *Swin-Transformer* | 87.8 |

**Table S3.** Performance metrics for classification model: equation and definition.

| **Metric** | **Equation** |
| --- | --- |
| True Positive (TP) | TP = Number of items correctly predicted as positive |
| True Negative (TN) | TN = Number of items correctly predicted as negative |
| False Positive (FP) | FP = Number of items incorrectly predicted as positive |
| False Negative (FN) | FN = Number of items incorrectly predicted as negative |
| Accuracy | Accuracy = (TP + TN) / (TP + TN + FP + FN) |
| Precision | Precision = TP / (TP + FP) |
| Recall | Recall = TP / (TP + FN) |
| F1-score | F1 = 2 * (Precision * Recall) / (Precision + Recall) |
